# Successive Orthorhombic Distortions in Kagome Metals by Molecular Orbital Formation

**DOI:** 10.1002/adma.202513015

**Published:** 2025-12-05

**Authors:** Ryo Misawa, Shunsuke Kitou, Rinsuke Yamada, Tobi Gaggl, Ryota Nakano, Yudai Shibata, Yoshihiro Okamura, Markus Kriener, Priya Ranjan Baral, Yuiga Nakamura, Yoshichika Ōnuki, Youtarou Takahashi, Taka‐hisa Arima, Milena Jovanovic, Leslie M. Schoop, Max Hirschberger

**Affiliations:** ^1^ Department of Applied Physics and Quantum‐Phase Electronics Center (QPEC) The University of Tokyo Bunkyo‐ku Tokyo 113‐8656 Japan; ^2^ Department of Advanced Materials Science The University of Tokyo Kashiwa Chiba 277‐8561 Japan; ^3^ RIKEN Center for Emergent Matter Science (CEMS) Wako Saitama 351‐0198 Japan; ^4^ Japan Synchrotron Radiation Research Institute (JASRI) SPring‐8 Hyogo 679‐5198 Japan; ^5^ Department of Chemistry North Carolina State University Raleigh NC 27695‐8204 USA; ^6^ Department of Chemistry Princeton University Princeton New Jersey 08544 USA

**Keywords:** diffuse scattering, kagome metal, molecular orbital, orthorhombic distortion, short‐range order

## Abstract

The kagome lattice, with its inherent frustration, hosts a plethora of exotic phenomena, including the emergence of 3 ***q*** charge‐density‐wave order. The high rotational symmetry required to realize such an unconventional charge order is broken in many kagome materials by orthorhombic distortions at high temperature, the origin of which remains much less examined despite their ubiquity. In this study, synchrotron X‐ray diffraction reveals a structural phase transition from a parent hexagonal structure to an orthorhombic ground state, mediated by a critical regime with diffuse scattering in the prototypical kagome metals *R*Ru_3_Si_2_ (*R* = Nd, Pr). Structural analysis uncovers partially ordered bonds between kagome layers in the orthorhombic phases. Accordingly, a short‐range correlated dimer model on the kagome layers reproduces the diffuse scattering, with the short‐range order arising from competing structures induced by the geometrical frustration of the kagome lattice. The observations point to molecular orbital formation between Ru $4d_{z^{2}}$ orbitals as the driving force behind the transition, consistent with ab initio calculations. A framework based on electronegativity and a tolerance factor is proposed to evaluate the stability of the hexagonal phase in various kagome metals, guiding the design of highly symmetric materials.

## Graphical abstract

Orthorhombic distortions in kagome metals break the symmetry required for exotic charge order. Synchrotron X‐ray diffraction of NdRu_3_Si_2_ reveals successive orthorhombic transitions driven by molecular orbital formation between kagome layers. The frustrated kagome lattice hosts degenerate patterns of kagome dimers whose short‐range correlations produce diffuse scattering. A chemistry‐based framework provides design principles for realizing structurally pristine kagome metals.

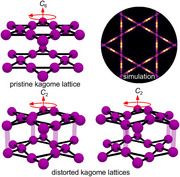

## 1 Introduction

The unique geometry of the kagome lattice, a network of corner‐sharing triangles, gives rise to characteristic electronic structures, namely, Dirac cones, flat bands (FB), and van Hove singularities (vHs).^[^ ^1^, ^2^ ^]^ Kagome metals can generally be categorized into four primary structural families, each distinguished by its specific stacking arrangement of triangular, honeycomb, and kagome lattices.^[^ ^3^, ^4^ ^]^ These families include the FeGe‐type (1‐1),^[^ ^5^ ^]^ KV_3_Sb_5_‐type (1‐3‐5),^[^ ^6^ ^]^ ScV_6_Sb_6_‐type (1‐6‐6),^[^ ^7^ ^]^ and LaRu_3_Si_2_‐type (1‐3‐2).^[^ ^8^, ^9^ ^]^ Each family exhibits distinct structural motifs, which, in turn, influence their electronic landscapes and resulting physical properties. Among these kagome metals, LaRu_3_Si_2_ stands out as a unique flat‐band host exhibiting the highest superconducting transition temperature of *T*_c_ ≈ 7 K.^[^ ^4^, ^8^, ^10^ ^]^ The superconducting state is proposed to be driven by mode‐selective coupling between kagome $d_{x^{2}-y^{2}}$ orbitals and kagome phonons.^[^ ^4^, ^11^ ^]^

In these broad classes of kagome metals, orthorhombic distortions are common and are often either suppressive of or accompanied by charge‐density‐wave (CDW) order—an emerging area of research. In fact, CDWs in kagome materials are of significant interest: a prominent example is the 3 ***q*** CDW order on the kagome lattice observed in *A*V_3_Sb_5_ (*A* = K, Rb, Cs),^[^ ^12^ ^]^ which may involve both rotational symmetry breaking and time‐reversal symmetry breaking well below the CDW transition.^[^ ^13^, ^14^, ^15^ ^]^ The electronic instability associated with vHs is considered a primary mechanism for the formation of this highly symmetric charge order.^[^ ^16^, ^17^, ^18^, ^19^, ^20^, ^21^ ^]^ On the other hand, the $\sqrt{3}\times \sqrt{3}$‐type CDW order observed in the 1‐6‐6 (*MT*_6_ *Z*_6_) family (*M* =  metallic element, *T* =  transition metal, and *Z* =  main group element) is among the most commonly encountered examples,^[^ ^7^, ^22^, ^23^ ^]^ which is also reported for LuNb_6_Sn_6_ and *T*Co_3_B_2_ (*T* = Zr, Hf).^[^ ^24^, ^25^ ^]^

**Figure 1:**
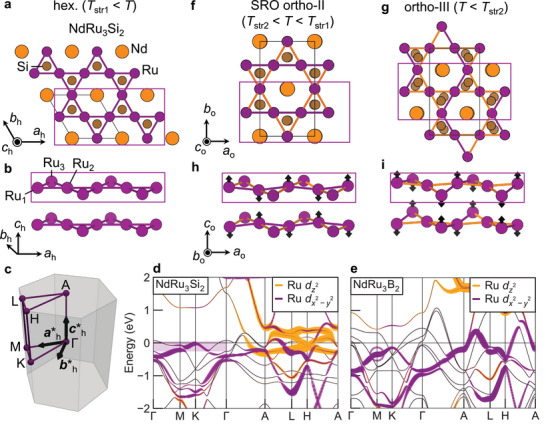
Successive orthorhombic distortions in kagome metals *R*Ru_3_Si_2_ (*R* = rare‐earth). a) Hexagonal structure in space group *P*6/*mmm* above *T*_str1_ = 760 K in NdRu_3_Si_2_. Ru atoms form the kagome lattice. b) Side view of kagome planes for the hexagonal structure. c) Hexagonal Brillouin zone with high‐symmetry points labeled. d,e) Band structures of NdRu_3_Si_2_ and NdRu_3_B_2_ with flat bands (purple area) and the van Hove singularity (purple ring) near the Fermi level in the hexagonal phase, respectively. Orange and purple colors represent $d_{z^{2}}$ and $d_{x^{2}-y^{2}}$ orbitals, respectively. f) Short‐range ordered (SRO) orthorhombic structure (ortho‐II, space group: *Ibmm*) of NdRu_3_Si_2_ between *T*_str1_ and *T*_str2_ = 720 K. Ru atoms dimerize along the *c*_o_ axis. The dimer configuration exhibits long‐range order along the *a*_o_ and *c*_o_ axes, but remains SRO along the *b*_o_ axis. Orange and purple colors indicate short and long Ru–Ru bonds, respectively. The Ru displacements are based on the structural model used in the diffuse‐scattering simulations shown in Figure 2a,b, as described in the Experimental Section. g) Orthorhombic structure (ortho‐III, space group: *Pbmm*) below *T*_str2_. Compared to ortho‐II in panel (f), the Ru–Ru dimers are recombined, as seen from the zigzag pattern of the purple bonds. Subscripts h and o on the unit cell axes denote the hexagonal and orthorhombic phases, respectively. h,i) Side views of kagome layers for ortho‐II and ortho‐III. Arrows indicate the direction of displacement. Purple regions correspond to those in panels (a),(f), and (g).

On the other hand, many kagome metals distort, often above room temperature, into orthorhombic or monoclinic structures with low symmetry. Within the *MT*_6_ *Z*_6_ family, versatile low‐symmetry structures are reported at room temperature.^[^ ^26^, ^27^ ^]^ The orthorhombic distortion is attributed to displacements of *M*‐*Z* chains along the *c* axis through phonon calculations.^[^ ^28^ ^]^ As for the 1‐1 family, FeGe is reported to undergo a monoclinic distortion below the magnetic transition, and subsequently increases its symmetry to orthorhombic upon entering the CDW phase.^[^ ^29^ ^]^ In the 1‐3‐2 family, all the known silicides exhibit orthorhombic structures at room temperature.^[^ ^30^, ^31^, ^32^, ^33^ ^]^ As compared to the undistorted hexagonal structure in **Figure** **1**a,b, the orthorhombic phases have a two‐times expanded *c* axis lattice constant. Despite the formation of a superstructure, this is a typical structural phase transition rather than a genuine CDW order, as discussed in this study. A recent example is LaRu_3_Si_2_, which crystallizes in the orthorhombic *Cccm* structure, hereafter referred to as ortho‐I, with two subsequent CDW transitions.^[^ ^9^, ^34^ ^]^ One of these is similar to the one in the recently discovered CsCr_3_Sb_5_, which exhibits superconductivity under pressure.^[^ ^35^ ^]^ So far, however, the crystal structures of the other *R*Ru_3_Si_2_ remain unknown. While ab initio calculations suggest soft phonon modes in the pristine hexagonal phase of LaRu_3_Si_2_,^[^ ^4^, ^9^ ^]^ the underlying origin of this type of orthorhombic structural instability remains unresolved—not only in this material, but across broader classes of kagome metals.

In this study, we choose *R*Ru_3_Si_2_ and *R*Ru_3_B_2_ (*R* = rare‐earth element) as prototypical kagome metals with kagome‐derived bands close to the Fermi energy. In the hexagonal Brillouin zone (Figure 1c), *R*Ru_3_Si_2_ exhibits kagome FB in the *k*_*z*_ = 0 plane (Figure 1d), while *R*Ru_3_B_2_ shows vHs at the *M* point (Figure 1e). We investigate structural stability by synchrotron X‐ray diffraction (XRD) using single crystals. In *R*Ru_3_Si_2_ (*R* = Nd, Pr), we observe the structural phase transition from the parent hexagonal structure (Figure 1a) to the orthorhombic structure (ortho‐III) (Figure 1g) via a short‐range correlated structure (ortho‐II) with diffuse scattering (Figure 1f). The low‐temperature structure is characterized by *c* axis doubling, similar to other orthorhombic kagome metals.^[^ ^30^, ^31^, ^32^, ^33^, ^36^ ^]^ We observe Ru–Ru dimerization along this axis (Figure 1h,i) and thus ascribe the diffuse scattering in the intermediate‐temperature range to short‐range order of kagome dimers. The short‐range ordered dimer model, incorporating competing structures, successfully captures the essential features of the diffuse scattering and highlights the role of geometrical frustration. Supported by electronic structure calculations, we attribute the origin of this phase transition to molecular orbital formation between the kagome $d_{z^{2}}$ orbitals. By comparing with *R*Ru_3_B_2_ (*R* = Pr, Gd, Lu), which preserve the ideal kagome lattice down to low temperatures, we propose a framework for assessing the structural chemistry of kagome metals. The present framework, based on electronegativity and atomic radii, may be applicable to a wide variety of kagome metals beyond the 1‐3‐2 systems. More broadly, our work also offers a guiding principle for the design of tailored kagome compounds with hexagonal symmetry and clean kagome bands.

## 2 Results

### 2.1 Sequence of Orthorhombic Distortions in *R*Ru_3_Si_2_

**Figure 2:**
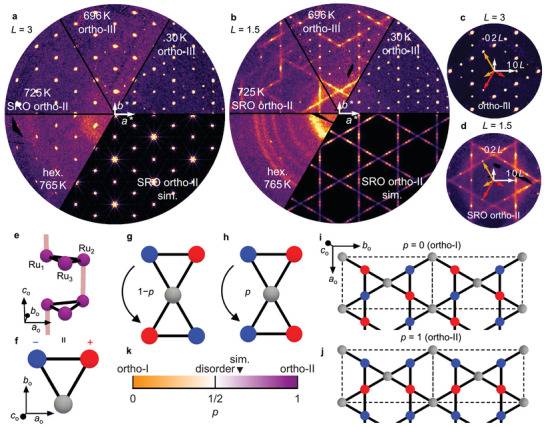
Temperature‐dependent reciprocal space map and modeling of the short‐range ordered structure in NdRu_3_Si_2_. a) XRD pattern in the *H* *K* 3 plane in the orthorhombic basis from four distinct phases: hexagonal (765 K), short‐range ordered (SRO) ortho‐II (725 K), ortho‐III with diffuse scattering (696 K), and ortho‐III (30 K). The simulated pattern corresponds to the SRO ortho‐II phase. Here, the Miller index *L* is defined relative to the undoubled *c* axis. b) XRD pattern in the *H* *K* 1.5 plane with simulated diffuse scattering. The data at 696 K in panels (a) and (b) are from a different sample. c,d) Close‐up views around 0 0 *L* clarifying the orthorhombic basis and the correspondence among orthorhombic bases for *L* = 3 (30 K, ortho‐III) and *L* = 1.5 planes (725 K, SRO ortho‐II), respectively. White arrows mark orthorhombic reciprocal lattice vectors; orange and red arrows are connected by a *C*_3_ rotation representing different orthorhombic domains. e) Schematic of kagome dimers in ortho‐II, corresponding to Figure 1h. Ru_1_ and Ru_2_ form dimers, colored in red, while Ru_3_ remains undimerized. f) 2D description of panel (e) with bonding and non‐bonding regions represented as charge‐rich (red) and charge‐poor (blue). The undimerized state of Ru_3_ is regarded as a neutral charge (gray). g,h) Two distorted kagome structures derived from panel (f). With probability *p*, the charge signs are the same between the two triangles and, with 1 − *p*, they are opposite. i,j) Long‐range ordered ortho‐I and ortho‐II when *p* = 0 and 1, respectively. Dashed lines indicate orthorhombic unit cells. k) Structural phase diagram as a function of *p*. When *p* = 1/2, ortho‐I and ortho‐II appear with equal probability, and the structure is completely disordered. 0 < *p* < 1/2 and 1/2 < *p* < 1 correspond to SRO ortho‐I (orange) and ortho‐II (purple), respectively. The intensity of colors represents the strength of structural correlation along the *b*_o_ axis. The black triangle indicates a point (*p* = 0.65) at which the diffraction patterns are calculated in panels (a,b).

We perform synchrotron XRD experiments on NdRu_3_Si_2_ and PrRu_3_Si_2_ to reveal their structural properties. Since they behave similarly, we focus on NdRu_3_Si_2_ in the main text. **Figure** **2**a,b show the XRD data on the *H* *K* 3 and *H* *K* 1.5 planes, respectively. We visualize the reciprocal space using an orthorhombic basis defined by the real‐space vectors ***a***_o_ = ***a***_h_, ***b***_o_ = ***a***_h_ + 2 ***b***_h,_ and ***c***_o_ = and ***c***_h_.

Upon cooling below *T*_str2_ = 720 K, we observe a new set of Bragg reflections in both *L* = integer and half‐integer planes, indicating a doubling of the *c* axis. This structure persists down to the lowest measured temperature of 30 K. A systematic absence of 0 *K* *L* reflections with *K* = odd indicates the presence of a *b*‐glide mirror perpendicular to the *a*_o_ axis. Satellite reflections, such as 1 0 3, can be indexed by three *C*_3_‐symmetric domains of the primitive orthorhombic cell (Figure 2c). On this basis, we refine the structure at 30 K assuming the orthorhombic *Pbmm* space group (ortho‐III), achieving a good agreement between model and experimental data when the volume fraction of three domains is 0.3091(3), 0.03197(12), and 0.6589(3).^[^ ^37^ ^]^ We note that the resolution of the 2D detector used in this study does not allow us to observe the splitting of Bragg reflections associated with the orthorhombic distortion. However, the XRD intensity distribution of the satellite reflections confirms the breaking of six‐fold symmetry, similar to LaRu_3_Si_2_.^[^ ^9^ ^]^ Consistent with our structure refinement, optical birefringence measurements visualize the three orthorhombic domains on a single crystal of NdRu_3_Si_2_.^[^ ^37^ ^]^

We also perform XRD on *R*Ru_3_B_2_ (*R* = Pr, Gd, and Lu) to compare the structural phase transition with *R*Ru_3_Si_2_. These materials do not show any structural transition from *P*6/*mmm* down to 30 K.^[^ ^37^ ^]^

### 2.2 Short‐Range Correlation of Kagome Dimers

In the intermediate regime between *T*_str1_ = 760 K and *T*_str2_, we observe the diffuse scattering in the *L* = half‐integer planes as well as orthorhombic Bragg reflections. First, ignoring the weak diffuse scattering intensity and focusing on the Bragg reflections, we find a systematic absence of 0 *K* *L* reflections with *K* = odd and *H* *K* *L* reflections with *H* + *K* + 2*L* = odd (Figure 2a,b). Close‐up views around the 0 0 1.5 position are shown in Figure 2d, providing a clear illustration of these extinction conditions. From these observations, we assign the average structure to the *Ibmm* space group (ortho‐II), with refined domain ratios of 0.187(7), 0.182(5), and 0.631(6).^[^ ^37^ ^]^

We then analyze the diffuse scattering, which is subsequently attributed to the short‐range correlation of Ru–Ru dimers observed in the low‐temperature phases. Key features are i) its presence along the *b*_o_ axis with *C*_3_ symmetric directions arising from the other domains, and ii) its absence in the *L* = integer or *H* = even planes. On one hand, i) suggests that the structure is long‐range correlated in the *a*_o_ *c*_o_ plane but not along the *b*_o_ axis in real space. On the other hand, ii) indicates that two sublattices connected by a 1/2‐translation along the *c*_o_ axis or *a*_o_ axis must have opposite displacements. From the refinement of the average structure in Figure 1f,h, the conditions on sublattices are satisfied by two Ru atoms, Ru_1_ and Ru_2_ (Figure 2e), which dimerize between adjacent kagome layers. To account for the diffuse scattering, we thus build a dimer model with short‐range correlations on the kagome Ru sites.

As shown in Figure 2e, the triangular Ru layers can be mapped from interlayer (non‐)bonding states onto charge‐rich (poor) sites, as indicated by red (blue) balls (Figure 2f). The undimerized Ru_3_ site is regarded as charge‐neutral, which is represented in gray. Because bonding and non‐bonding states are staggered along the *c*_o_ axis (Figure 2e), the charge signs also reverse between neighboring layers. This mapping naturally hints at two ways to construct a distorted kagome lattice: either flipping (Figure 2g) or repeating the charge signs on the next inverted triangle (Figure 2h), which are nearly degenerate due to geometrical frustration of the kagome lattice. We assign the probability of the latter as *p*, with that of the former then given as 1 − *p*. The limiting cases of *p* = 0 and *p* = 1 correspond to long‐range ordered ortho‐I and ortho‐II structures, respectively (Figure 2i,j). On the other hand, the structure is disordered when *p* = 1/2. The coexistence of ortho‐II‐type Bragg spots and the diffuse scattering in NdRu_3_Si_2_ suggests that it belongs to the short‐range correlated structure with 1/2 < *p* < 1, where ortho‐II dominates but ortho‐I remains locally present (Figure 2k). We thus simulate diffraction patterns with *p* = 0.65 (Figure 2a,b), reproducing the experimental observations for NdRu_3_Si_2_. By contrast, *p* = 1/2 (disorder) and 0 < *p* < 1/2 (short‐range ordered ortho‐I) correspond to two intermediate regimes with diffuse scattering observed in LaRu_3_Si_2_, also captured within the same scheme.^[^ ^37^ ^]^

In this framework, the observed short‐range order reflects geometrical frustration of the kagome lattice, which renders ortho‐I and ortho‐II nearly degenerate. The resulting competition gives rise to short‐range correlations involving the locally coexisting structure.

### 2.3 Long‐Range Orthorhombic Phase Stabilized by Dimer Recombination

Upon cooling, the short‐range correlation is suppressed through a recombination of the dimers and a concomitant reduction in entropy. Below *T*_str2_, new diffraction spots, consistent with ortho‐III, gradually emerge. Nevertheless, the diffuse scattering from remnants of the short‐range correlated structure persists until around 660 K.^[^ ^37^ ^]^ The eventual transition to the long‐range ordered ortho‐III is driven by a reorganization of dimer bonds: one of the Ru_1_–Ru_1_ or Ru_2_–Ru_2_ dimers is replaced by new dimers involving the third, central Ru atom (Ru_3_) (Figure 1i). Importantly, this reorganization is enabled by the presence of three Ru sublattices within each kagome unit, a feature unique to the kagome lattice. Such triadic connectivity allows for a rich dimer landscape and plays a key role in the transition mechanism.

### 2.4 Critical Behavior of XRD Intensities

**Figure 3:**
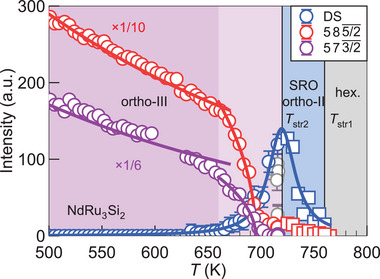
Critical behavior and effects of the Debye–Waller factor in single‐crystal X‐ray diffraction intensities of NdRu_3_Si_2_. Gray, blue, and purple regions indicate hexagonal (hex.), short‐range ordered (SRO) ortho‐II, and ortho‐III phases. In the light purple area, the SRO structure is gradually suppressed as ortho‐III stabilizes. Blue, red, and purple open circles denote diffuse scattering, $5\;8\;\bar{5/2}$, and $5\;7\;\bar{3/2}$ reflections, respectively, in an orthorhombic basis without *c* axis doubling. The diffuse scattering is measured at $1/4\;9/4\;\bar{5/2}$, where no Bragg reflection is expected. The blue line is a guide to the eye. The red and purple lines are fits to the Bragg intensities with a Debye–Waller factor and scaling functions. Open squares are from a different dataset, as explained in the Experimental Section. Clear outliers are colored gray.

To track the successive structural transitions, we perform temperature‐dependent XRD. In **Figure** **3**, we show the diffuse scattering intensity in blue and the Bragg reflections characterizing the ortho‐II and ortho‐III structures in red and purple, respectively. The diffuse scattering starts to evolve at *T*_str1_, at the same time as the ortho‐II‐type Bragg reflections. It takes a maximum at *T*_str2_, from which it decreases and completely disappears at around 660 K – while the intensity of ortho‐III‐type Bragg reflections gradually increases.

The temperature dependence of the Bragg intensities well below the critical temperatures is entirely attributed to the Debye‐Waller factor, *I* = *I*_0_exp (− 2*W*), with $W=3\hbar^{2}{\left|G\right|}^{2}T/\;\left(2Mk_{B}\Theta_{D}^{2}\right)$ where **G**, *M*, Θ_D_ are the reciprocal lattice vector, the mass of an atom (Ru), and the Debye temperature. From this equation and a fit to the temperature dependent intensity of $5\;8\;\bar{5/2}$ and $5\;7\;\bar{3/2}$, we estimate Θ_D_ = 154  and 182 K, respectively, which are comparable to Θ_D_ = 194 K from the analysis of the low‐temperature specific heat in LaRu_3_Si_2_ when including a correction for electronic correlations.^[^ ^38^ ^]^

In the intermediate temperature range, critical fluctuations play a role. For the diffuse scattering, we observe a diverging behavior of the intensity *I*(*T*) toward *T*_str2_. The diffuse scattering is related to the susceptibility, and thus this critical behavior follows the power law *I*∝|*t*|^−*γ*^, where *t* is the reduced temperature defined as *t* = (*T* − *T*_str2_)/*T*_str2_ and γ is the critical exponent of the susceptibility.^[^ ^39^ ^]^ Fitting to this functional form, we obtain γ = 1.1(2), consistent with the mean‐field value (γ = 1.0). As for the Bragg reflections, their intensities gradually develop and then follow the scaling behavior of an order parameter with the mean‐field critical exponent β = 1/2. Our differential scanning calorimetry measurements confirm an anomaly at 722 K,^[^ ^37^ ^]^ consistent with *T*_str2_ in XRD.

### 2.5 Molecular Orbital Formation as the Origin of Successive Orthorhombic Distortions

**Figure 4:**
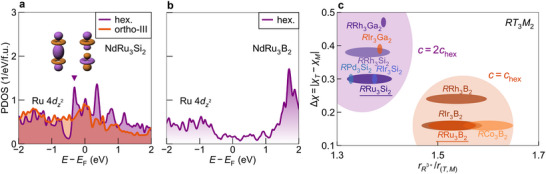
Molecular orbital formation of kagome $d_{z^{2}}$ orbitals and generalization to *RT*_3_ *M*_2_ kagome metals (*R* = rare‐earth element, *T* = transition metal, *M* = main‐group element). a,b) Partial density of states (PDOS) of Ru‐4$d_{z^{2}}$ orbitals for NdRu_3_Si_2_ and NdRu_3_B_2_, calculated using density functional theory. Purple and orange colors represent hexagonal and ortho‐III phases, respectively. The purple triangle indicates the PDOS peak, which is removed by molecular orbital formation between kagome layers. Schematics of bonding and anti‐bonding states are shown as insets. c) Classification of *RT*_3_ *M*_2_ kagome metals based on electronegativity difference and a tolerance factor. χ_*T*_ (χ_*M*_) is the electronegativity of *T* (of *M*), and Δχ = |χ_*T*_ − χ_*M*_| is the difference between them. $r_{R^{3+}}$ and *r*_(*T*, *M*)_ are the ionic radii of *R* ^3 +^ and the average atomic radius of *T* and *M*, respectively. The tolerance factor is defined by their ratio. The orange region includes hexagonal and monoclinic structures with the lattice constant *c* equal to that of the pristine hexagonal phase (*c*_hex_), while the purple area contains orthorhombic structures with *c* = 2*c*_hex_.

So far, we have revealed the successive orthorhombic distortions in *R*Ru_3_Si_2_ driven by the consecutive dimerization of kagome Ru atoms and the absence of such a transition in *R*Ru_3_B_2_. The dimers along the *c*_o_ axis in *R*Ru_3_Si_2_ imply molecular orbital formation of the kagome $d_{z^{2}}$ orbitals. To validate this scenario, we perform ab initio calculations of the partial density of states (PDOS) of Ru‐$d_{z^{2}}$ for NdRu_3_Si_2_ and NdRu_3_B_2_. We find that the PDOS peak in the hexagonal phase of NdRu_3_Si_2_, located near the Fermi level, is removed after the structural transition to ortho‐III, as illustrated in **Figure** **4**a. This is consistent with the molecular orbital formation of kagome $d_{z^{2}}$ orbitals. A competing scenario for the high‐temperature phase transition is Fermi surface nesting with a related electronic instability. This is excluded from first‐principle calculations for the Fermi surface of NdRu_3_Si_2_.^[^ ^37^ ^]^ On the other hand, almost no $4d_{z^{2}}$ PDOS is observed for NdRu_3_B_2_ around the Fermi energy (Figure 4b]. These features are also evident in the orbital projected band structure of NdRu_3_Si_2_ and NdRu_3_B_2_, depicted in Figure 1d,e, respectively.

We attribute the difference in the Ru‐$d_{z^{2}}$ orbitals' contribution to the variation in covalent bonding strength between Ru and the surrounding B or Si atoms. Compared to silicon's electronegativity (χ_Si_ = 1.9), boron's (χ_B_ = 2.0) is closer to that of ruthenium (χ_Ru_ = 2.2), and thus Ru‐$d_{z^{2}}$ orbitals form stronger covalent bonds with unoccupied B‐*p* orbitals. This bonding interaction pushes the Ru‐$d_{z^{2}}$ states away from the Fermi level in *R*Ru_3_B_2_. In contrast, in the silicon analog, the weaker bonding between Ru‐$d_{z^{2}}$ and Si‐*p* orbitals allows the $d_{z^{2}}$ states to remain near the Fermi level, making them available for dimer formation. Comparison of the PDOS for Ru‐$d_{z^{2}}$ and B‐/Si‐*p*_*z*_ orbitals also supports this scenario.^[^ ^37^ ^]^ We compare the band structures for the hexagonal and ortho‐III structures in the Supporting Information.^[^ ^37^ ^]^

### 2.6 Molecular Orbital Formation in 1‐3‐2 Kagome Metals

Generalizing to the entire 1‐3‐2 family of kagome metals, hexagonal structures are favored when i) the difference in electronegativity Δχ between the transition metal (*T*) and main‐group element (*M*), defined as Δχ = |χ_*T*_ − χ_*M*_|, is small and ii) the tolerance factor, defined as the ratio of the ionic radius of *R* ^3 +^ and the average radius of *T* and *M* atoms, expressed as $t=r_{R}^{3+}/r_{\left(T,M\right)}$, is moderately large. For the 1‐3‐2 family, we have *r*_(*T*, *M*)_ = (3*r*_*T*_ + 2*r*_*M*_)/5. Here, Δχ measures the availability of *T*‐site $d_{z^{2}}$ orbitals near the Fermi level, which is essential for the kagome dimer formation, and *t* quantifies the phase stability, explicitly incorporating the influence of rare‐earth ionic radii. This factor is crucial not only for assessing structural stability but also for distinguishing variations within the rare‐earth series, which cannot be captured by Δχ. We plot these two parameters for various 1‐3‐2 kagome metals in Figure 4c.^[^ ^30^, ^31^, ^32^, ^33^, ^36^, ^40^, ^41^, ^42^, ^43^, ^44^, ^45^, ^46^ ^]^ In the *AB*_5_‐type (1–5) family, which is isostructural to the 1–3–2 family upon replacing the *M* site with *T* atoms, the stability range has been shown to lie within 1.30 < *t* = *r*_*A*_/*r*_*B*_ < 1.77.^[^ ^47^ ^]^ Figure 4c confirms similar behavior in the 1‐3‐2 systems. The plot demonstrates that the borides have lower Δχ and larger *t* so that they commonly do not show the *c* axis doubling or orthorhombic distortions, while the opposite is true for all silicides and gallides. As silicides and gallides are in close proximity to the lower limit of *t*, distorted structures may be preferred. The gallide LaIr_3_Ga_2_ stands out as an exceptional hexagonal system, likely due to its strong two‐dimensionality: *c*/*a* = 0.7.

Focusing on *R*Ru_3_Si_2_, the crystal symmetry and structural stability systematically decrease with increasing rare‐earth atomic number. While LaRu_3_Si_2_ adopts the ortho‐I (*Cccm*) phase, the heavier *R*Ru_3_Si_2_ compounds (Pr, Nd) crystallize in the lower‐symmetry ortho‐III (*Pbmm*) structure. This trend is corroborated by our differential scanning calorimetry results, which show that *T*_str2_ rises with heavier rare‐earth elements in *R*Ru_3_Si_2_.^[^ ^37^ ^]^ The reduction in ionic radius with increasing atomic number pushes the system toward the lower limit of the tolerance factor, thereby favoring the less symmetric phase. In contrast, other silicides such as *R*Rh_3_Si_2_ stabilize in ortho‐II. This structure is metastable in *R*Ru_3_Si_2_, and it transforms into ortho‐III through recombination of kagome dimers (Figure 1h,i). Such behavior likely depends on subtle variations in crystal parameters that tune the competition between lattice deformation energy and the energy gain from molecular‐orbital formation.

We note that a previous study evaluated the effects of the electronegativity difference between *A* and *B* in the 1–5 systems, concluding that it does not have a major impact on phase stability.^[^ ^47^ ^]^ However, its impact on orthorhombic distortions was not discussed. Indeed, our focus is on the electronegativity difference between the *T* and *M* sites, which are, in contrast, occupied by the same element in the 1–5 family.

## 3 Conclusion

In summary, we observe a sequence of orthorhombic structural phase transitions in *R*Ru_3_Si_2_ and its absence in *R*Ru_3_B_2_, utilizing synchrotron XRD. Through modeling of diffuse scattering, precise structural refinement, and ab initio calculations, we attribute the orthorhombic distortions to molecular orbital formation of kagome $d_{z^{2}}$ orbitals. This behavior appears to be a common feature of 1‐3‐2 kagome metals, as we find that the structural stability is correlated with the electronegativity difference between a transition metal and a main‐group element, as well as the tolerance factor defined by the ionic and atomic radius ratio.

As a precursor of charge‐density‐wave transitions, different types of diffuse scattering were reported in FeGe and ScV_6_Sn_6_.^[^ ^22^, ^23^, ^48^ ^]^ However, their origin is different from *R*Ru_3_Si_2_: for the former, it is Ge atoms on the triangular lattice that dimerize, while for the latter, it is likely a 1D Sc‐Sn chain irrelevant to the kagome lattice. Our short‐range correlated dimer model thus stands out by describing a collective instability inherent to the kagome lattice, where the three distinct sublattices generate a diverse landscape of competing dimer configurations.

Broadly speaking, a similar phenomenology emerges in spin systems, as our charge model is mapped directly onto the kagome Ising model. The ortho‐I, ortho‐II, and ortho‐III phases, featuring an unbonded site (neutral charge) equivalent to a free spin (Figures 1i, 2i,j], realize partial bond order as an example of partial order arising from geometrical frustration. Notably, the exact phase diagram of the kagome Ising model predicts such partial order in some of its ground states.^[^ ^49^ ^]^ Experimentally, partial order on the kagome lattice has been realized in spin systems: in intermetallic compounds with out‐of‐plane spin anisotropy^[^ ^50^ ^]^ and, more recently, in kagome spin‐ice systems with in‐plane anisotropy.^[^ ^51^, ^52^ ^]^ Our work, therefore, underscores the universality of geometrical frustration, forming a bridge between spin systems and the problem of structural instabilities or chemical bonding.

Beyond the 1–3–2 systems, orthorhombic kagome lattice instabilities are likely relevant across a wider set of kagome metals.^[^ ^37^ ^]^ In the 1–1 family, for example, the distance between two consecutive triangular layers is the same as that between two kagome layers, suggesting possible competing structural instabilities—those associated with the kagome lattice, similar to the present instabilities in the 1–3–2 family, and those of the triangular lattice, as reported for FeGe. Because structural instabilities in the 1–1 family remain scarcely studied, further exploration is needed to evaluate the broader applicability of our framework.

The chemistry‐based framework proposed here provides a foundation for designing kagome metals resilient against high‐temperature structural instabilities. By stabilizing clean kagome bands, such materials can host the full range of topological and correlated phenomena anticipated in kagome systems—including Dirac and Weyl semimetals,^[^ ^53^, ^54^, ^55^ ^]^ topological flat bands,^[^ ^2^, ^56^ ^]^ and instabilities associated with van Hove singularities, which can give rise to electronically driven orders such as the 3 ***q*** CDW order and nematic phases.^[^ ^12^, ^16^, ^17^, ^18^, ^19^, ^20^, ^21^ ^]^ In this way, our study establishes both a conceptual understanding of orthorhombic instabilities and a practical route toward realizing quantum phases of matter in kagome metals.

## 4 Experimental Section

### Crystal Growth

*R*Ru_3_Si_2_ and *R*Ru_3_B_2_ were synthesized by the arc melting technique in a high‐purity argon atmosphere. For *R*Ru_3_Si_2_, to avoid the competing magnetic impurity *R*Ru_2_Si_2_, excess Ru was added to the starting materials.^[^ ^8^ ^]^ Increasing excess Ru was required to obtain *R*Ru_3_Si_2_ with heavier rare‐earth elements. *R*Ru_3_B_2_ was melted from a stoichiometric combination of elements. Small single crystals, approximately 50 µm in size and suitable for XRD measurements, were obtained by mechanical fragmentation of the crystals. A 1 mm^3^ single crystal of NdRu_3_Si_2_, with around 15% of Ru impurity, for optical measurements was found from an ingot after the Czochralski growth.

### Single Crystal X‐Ray Diffraction

Synchrotron XRD was performed at BL02B1 of the synchrotron X‐ray facility SPring‐8 (Japan). Diffraction patterns were recorded using a CdTe PILATUS area detector. Temperature was controlled using N_2_ gas flow above 100 K and H_2_ gas flow below 100 K. Integrated intensities were collected in the CrysAlisPro program.^[^ ^57^ ^]^ Equivalent reflections were averaged, and structure parameters were refined in Jana2006.^[^ ^58^ ^]^ In the space group descriptions (e.g., *Ibmm*), the axes were retained as in the pristine structure and were not transformed to the conventional setting (e.g., *Imma*) to facilitate direct comparison. In Figure 3, the open circles represent data from one fixed frame during a temperature scan, while the squares correspond to intensities from the reconstructed reciprocal space in a dataset with an extended momentum scan. The latter were normalized using the intensity of a Bragg reflection common to both datasets. Crystal structures were drawn by VESTA.^[^ ^59^ ^]^

### Modeling of Diffuse Scattering

Diffuse scattering was modeled by considering the short‐range order of Ru atoms in the ortho‐II phase. The model assumed static, spatially short‐range correlations of kagome dimers and excluded thermal or phonon effects, which were not necessary to explain the observations. Along the *b*_o_ axis, the charge signs on a triangle were kept with the probability of *p* and reversed with that of 1 − *p*, constructing a supercell comprising *L*_*x*_ × *L*_*y*_ × *L*_*z*_ = 50×50×1  orthorhombic unit cells, each containing 12 Ru atoms. The displacement of Ru atoms along the *c* axis was denoted by δ. The average displacement was then given by δ_avg_ = |*p*δ − (1 − *p*)δ| = |2*p* − 1|δ. This corresponds to the value obtained from refinement of the average ortho‐II structure when only Bragg reflections were considered. At 725 K, δ_avg_ = 0.00677, giving δ = 0.0226 with *p* = 0.65, which is shown in Figure 1f,h and was used in the simulations. To mitigate finite‐size effects, the mesh spacing was set to 2π/*a*_o_ *L*_*x*_ (Å) and 2π/*b*_o_ *L*_*y*_ (Å) along the *k*_*x*_ and *k*_*y*_ directions, respectively. This resulted in a 565 × 979 mesh for computing reciprocal space maps at *L* = 1.5 and 3. To suppress unphysical coherence across the entire crystal, intensities were calculated over a randomly sampled volume of 10 × 10 × 1 unit cells, and the results were averaged over 50 samples. The total diffraction pattern was obtained by summing the intensities from three orthorhombic domains. For simplicity, equal occupation of domains was assumed. The atomic form factor of Ru was included in the calculation using the Cromer–Mann Gaussian parametrization. To enable direct comparison, the calculated diffraction patterns were linearly rescaled to the experimental mesh, as shown in Figure 2a,b. Contributions from the other elements were not found to be necessary to reproduce the experimentally observed diffuse scattering.

### Specific Heat Measurement

Differential scanning calorimetry (DSC) was performed over the temperature range of 300  to 850 K using a simultaneous thermal analyzer (STA 449 F1 TG‐DSC, Netzsch) under a nitrogen atmosphere. DSC signals were then converted to specific heat.^[^ ^37^ ^]^

### First‐Principles Calculations

First‐principles calculations were performed using the Vienna Ab initio Simulation Package (VASP) 6.4 with the Strongly Constrained and Appropriately Normed (SCAN) meta‐generalized gradient approximation for the exchange‐correlation functional.^[^ ^60^ ^]^ SCAN meta‐GGA is known to capture correlation effects, and the Hubbard *U* was not included in the calculations. Regarding the Ru *d* orbitals in particular, they play a central role in the chemical bonding within the lattice,^[^ ^61^ ^]^ and imposing their localization through *U* correction would therefore be unphysical. The projector augmented‐wave (PAW) pseudopotentials employed correspond to Nd_3_, Ru, Si, and B.^[^ ^62^, ^63^ ^]^ The Nd_3_ PAW pseudopotential creates a +3 cation and places the remaining *f* electrons into the frozen core, removing the need for localizing them using Hubbard *U* correction. Γ‐centered 16 × 16 × 16 and 16 × 10 × 8 Monkhorst–Pack grids were used for sampling the Brillouin zone of NdRu_3_Si_2_ in the hexagonal and ortho‐III phases, respectively, while a 15 × 15 × 19 grid was used for NdRu_3_B_2_. Spin‐orbit coupling, as implemented in VASP, was included in all calculations.^[^ ^64^ ^]^ Structural relaxations were performed using the residual minimization method with direct inversion in the iterative subspace (RMM‐DIIS), employing a step size of 0.25.^[^ ^65^ ^]^

### Optical Birefringence Measurement

Optical birefringence was measured for real‐space observation of orthorhombic domains using an optical microscope with polarizer and analyzer plates.^[^ ^66^ ^]^ An LED light source (M970L4, Thorlabs) with a center wavelength of 970 nm and a bandwidth of approximately 50 nm was used. Linearly polarized, near‐infrared collimated light was directed onto the sample whose surface was perpendicular to the *c* axis. The reflected light was then collected and passed through an analyzer before reaching a CMOS camera (CS505MO, Thorlabs), which captured a polarization‐resolved image of the sample. The incident polarization angle dependence of the polarization rotation angle was measured by rotating the incident polarization while fixing the sample.

## Conflict of Interest

The authors declare no conflict of interest.

## Supporting information

Supporting Information

## Data Availability

The data that support the findings of this study are available from the corresponding author upon reasonable request.
