# Supplementary material for: Successive Orthorhombic Distortions in Kagome Metals by Molecular Orbital Formation
Source: Adv Mater. 2025 Dec 5;38(8):e13015. doi: 10.1002/adma.202513015 (PMC12878805; doi:10.1002/adma.202513015)
Supplement: Supplementary file 1 — Supporting Information [file ADMA-38-e13015-s001.pdf]

ADVANCED MATERIALS

Supporting Information

for *Adv. Mater.*, DOI 10.1002/adma.202513015

Successive Orthorhombic Distortions in Kagome Metals by Molecular Orbital Formation

Ryo Misawa, Shunsuke Kitou, Rinsuke Yamada, Tobi Gaggl, Ryota Nakano, Yudai Shibata, Yoshihiro Okamura, Markus Kriener, Priya Ranjan Baral, Yuiga Nakamura, Yoshichika Ōnuki, Youtarou Takahashi, Taka-hisa Arima, Milena Jovanovic, Leslie M. Schoop and Max Hirschberger**

CRYSTAL STRUCTURE REFINEMENT OF NDRU$_3$SI$_2$

Results of the crystal structure refinement for $NdRu_3Si_2$ including structure parameters and twin information are provided in Tables S1-S4. Since the diffraction patterns do not show any observable peak splitting associated with orthorhombic domains, we refine the orthorhombic structures under the constraint $b = \sqrt{3}a$.

TABLE S1. Summary of crystallographic data for $NdRu_3Si_2$. Z and $F(000)$ represent formula units per unit cell and the structure factor at the origin, respectively.

Space group	$P6/mmm$ (No. 191)	$Ibmm$ (No. 74)	$Pbmm$ (No. 51)
Wavelength (Å)	0.30868	0.30868	0.30868
Temperature (K)	765	725	30
a (Å)	5.6402(4)	5.6405(2)	5.60470(10)
b (Å)	5.6402(4)	$\sqrt{3}a$	$\sqrt{3}a$
c (Å)	3.5970(4)	7.1934(2)	7.1297(2)
α (°)	90	90	90
β (°)	90	90	90
γ (°)	120	90	90
Cell volume (Å^3)	99.097(15)	396.40(2)	387.914(15)
Z	1	4	4
$F(000)$	220	880	880
Density (g/cm^3)	8.4409	8.4475	8.6253
Resolution limit (Å)	0.71	0.50	0.28
Reflections measured	20172	16137	22867
Unique reflections	79	2208	22867
Completeness (%)	98	98	98
R_1 ($I > 3\sigma$ / all)	3.09%/3.20%	4.91%/6.84%	3.09%/3.85%
wR_2 ($I > 3\sigma$ / all)	5.19%/5.27%	7.45%/7.55%	3.92%/3.94%
GOF ($I > 3\sigma$ / all)	2.34/2.32	4.20/3.02	2.19/1.74

TABLE S2. Atomic positions and displacement parameters for $NdRu_3Si_2$.

Atom	x	y	z	U_{eq} (Å^2)	Site Mult.	Site Sym.	Occ.
			hex. ($P6/mmm$, 765 K)				
Nd1	1	0	1	0.0240(6)	1	$6/mmm$	1
Ru1	1/2	0	1/2	0.0276(6)	3	mmm	1
Si1	2/3	1/3	0	0.040(2)	2	$-6m2$	1
			ortho-II ($Ibmm$, 725 K)				
Nd1	0.00387(8)	0	1/4	0.02078(16)	4	$mm2$	1
Ru1	1	1/2	1/2	0.0251(3)	4	$2/m..$	1
Ru2	3/4	3/4	0.50677(5)	0.02311(16)	8	.2.	1
Si1	0.5107(4)	0.8342(6)	1/4	0.0347(10)	8	$.m.$	1
			ortho-III ($Pbmm$, 30 K)				
Nd1	0.737556(15)	1/4	0	0.001635(10)	2	$mm2$	1
Nd2	0.756577(13)	1/4	1/2	0.001614(11)	2	$mm2$	1
Ru1	1/2	0	0.218780(10)	0.001674(9)	4	.2.	1
Ru2	1	0	0.253070(11)	0.001958(9)	4	.2.	1
Ru3	1.242111(13)	1/4	0.279766(12)	0.001620(10)	4	$m..$	1
Si1	0.22916(7)	0.10718(4)	0	0.00263(5)	4	$.m.$	1
Si2	0.28381(7)	0.06377(4)	1/2	0.00256(5)	4	$.m.$	1

SINGLE-CRYSTAL X-RAY DIFFRACTION FOR PRRU$_3$SI$_2$ AND RRU$_3$B$_2$

Figure S1 illustrates the reconstructed reciprocal space map of $PrRu_3Si_2$ at 720 K **a** and 30 K **b**. This confirms the short-range correlated ortho-II ($Ibmm$) and ortho-III ($Pbmm$) phases, similar to $NdRu_3Si_2$. Figure S2 shows the $L = 3$ plane for RRu_3B_2 (R=Pr, Gd, and Lu) at 30 K, which indicates the absence of a phase transition from the hexagonal structure.

TABLE S3. Anisotropic displacement parameters (Å^2) for $NdRu_3Si_2$.

Atom	U_{11}	U_{22}	U_{33}	U_{12}	U_{13}	U_{23}
hex. ($P6/mmm$, 765 K)						
Nd1	0.0244(8)	0.0244(8) = U_{11}	0.0232(10)	0.0122(4)	0	0
Ru1	0.0230(8)	0.0110(8)	0.0447(11)	0.0055(4)	0	0
Si1	0.052(3)	0.052(3) = U_{11}	0.015(3)	0.0262(14)	0	0
ortho-II ($Ibmm$, 725 K)						
Nd1	0.0202(3)	0.0210(3)	0.02110(18)	0	0	0
Ru1	0.0228(5)	0.0086(3)	0.0438(6)	0	−0.0011(2)	0
Ru2	0.0131(2)	0.0190(3)	0.0372(3)	0.0058(2)	0	0
Si1	0.044(2)	0.051(2)	0.0092(5)	−0.0041(9)	0	0
ortho-III ($Pbmm$, 30 K)						
Nd1	0.001140(15)	0.00190(2)	0.001864(18)	0	0	0
Nd2	0.001363(19)	0.00163(2)	0.001849(18)	0	0	0
Ru1	0.001181(15)	0.002073(16)	0.001768(15)	0.000541(12)	0	0
Ru2	0.001161(15)	0.002228(17)	0.002484(16)	−0.000499(13)	0	0
Ru3	0.001820(18)	0.001467(19)	0.001573(17)	0	0.000029(11)	0
Si1	0.00246(8)	0.00316(10)	0.00226(9)	0.00051(8)	0	0
Si2	0.00306(9)	0.00235(9)	0.00226(9)	−0.00003(7)	0	0

TABLE S4. Twin information of orthorhombic structures in $NdRu_3Si_2$. m_{ij} indicates an element of a twin matrix.

Twin ID	Mass Fraction	m_{11}	m_{12}	m_{13}	m_{21}	m_{22}	m_{23}	m_{31}	m_{32}	m_{33}
ortho-II ($Ibmm$, 725 K)										
1	0.187(7)	1	0	0	0	1	0	0	0	1
2	0.182(5)	−1/2	−3/2	0	1/2	−1/2	0	0	0	1
3	0.631(6)	−1/2	3/2	0	−1/2	−1/2	0	0	0	1
ortho-III ($Pbmm$, 30 K)										
1	0.3091(3)	1	0	0	0	1	0	0	0	1
2	0.03197(12)	−1/2	−3/2	0	1/2	−1/2	0	0	0	1
3	0.6589(3)	−1/2	3/2	0	−1/2	−1/2	0	0	0	1

DIFFERENTIAL SCANNING CALORIMETRY FOR $PrRu_3Si_2$

We obtain the specific heat $C(T)$ of $R\mathrm{Ru_3Si_2}$ at high temperature from differential scanning calorimetry (DSC). In the DSC measurement, we measure three times for each trial: (1) an empty sample holder (pan), (2) the pan with a reference sample, and (3) the pan with a sample. As a reference, sapphire with a known specific heat C_{ref} is used. The specific heat of the sample C_{sample} is then calculated as

$$C_{\mathrm{sample}} = \frac{\mathrm{DSC_{sample}} - \mathrm{DSC_{base}}}{\mathrm{DSC_{ref}} - \mathrm{DSC_{base}}} \cdot \frac{m_{\mathrm{ref}}}{m_{\mathrm{sample}}} C_{\mathrm{ref}} \tag{S1}$$

where DSC is the differential scanning calorimetry signal, m is the mass, and the subscript "base" denotes the value of the empty pan.

Figure S3**a** illustrates the high-temperature specific heat of $R\mathrm{Ru_3Si_2}$ (R=Pr, Nd, Sm, Er) obtained through DSC measurements. The anomaly around $700 \sim 800$ K corresponds to the phase transition from the hexagonal to the ortho-III phase, where the diffuse scattering intensity takes its maximum. The transition temperature follows an increasing trend for heavier rare-earth elements [Fig. S3**b**] and is above the measurement limit for R = Er. In our crystal growth, we also found that $R\mathrm{Ru_3Si_2}$ with heavier rare-earth elements are harder to synthesize.

REAL-SPACE OBSERVATION OF ORTHORHOMBIC DOMAINS

To confirm the orthorhombic symmetry in real space, we visualize the spatial distribution of the polarization rotation angle θ_{B} induced by the birefringence effect at 300 K, as illustrated in Fig. S4**a**. We take the 0-degree direction to be along the a^* axis. Figure S4**b** shows the spatial map of θ_{B} measured with incident polarization angles of 0, 120, and 240 degrees, respectively. Three distinct regions, colored red, blue, and green, exhibit different dependencies on the incident polarization angle. To clarify this, we analyze the polarization-angle dependence of θ_{B} in these regions

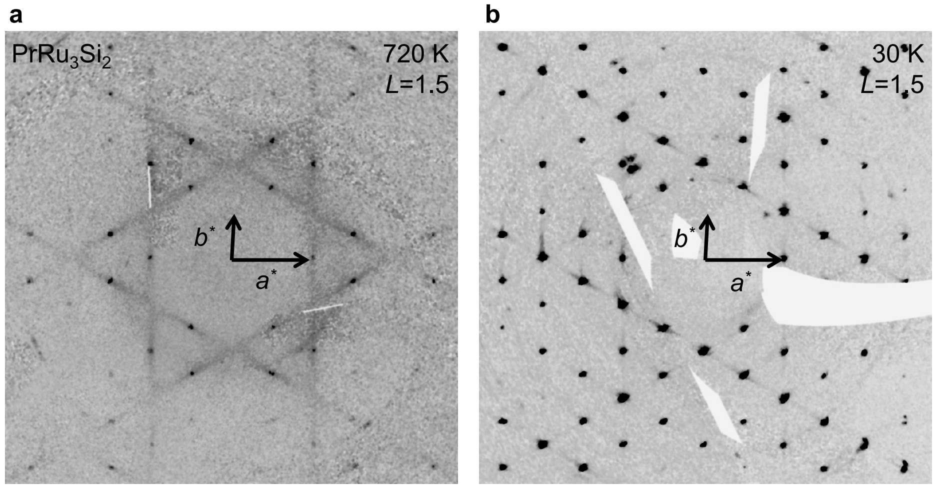

FIG. S1. **a**,**b** Reconstructed reciprocal space ($L = 1.5$) for $PrRu_3Si_2$ at 720 K (sample 1) and 30 K (sample 2), respectively. $PrRu_3Si_2$ exhibits the same successive orthorhombic distortions as $NdRu_3Si_2$.

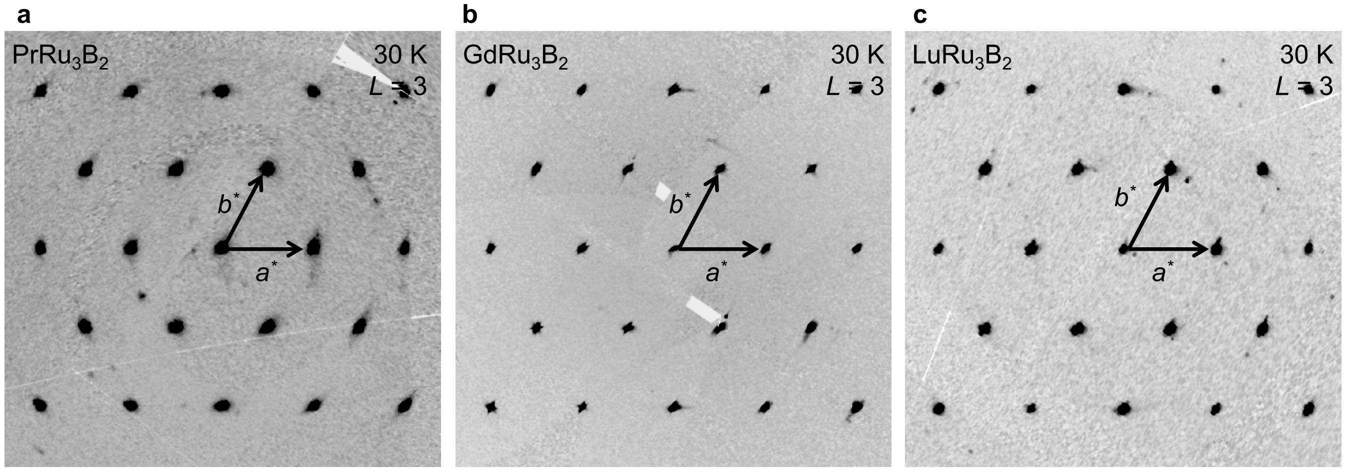

FIG. S2. Absence of a structural phase transition from the hexagonal $P6/mmm$ structure down to 30 K in RRu$_3$B$_2$ (R = Pr, Gd, and Lu). **a**,**b**,**c**, Reconstructed reciprocal space ($L = 3$) at 30 K for RRu$_3$B$_2$ with R = Pr, Gd, and Lu, respectively.

[Fig. S4**c**]. Each shows a clear twofold (C_2) symmetry, but the angular dependence is shifted by 120 degrees between the regions. This behavior is expected for three orthorhombic domains rotated by 120 degrees relative to one another. Importantly, in the blue region θ_B reaches a minimum near 75 degrees, corresponding to a direction 45 degrees from the crystallographic a axis when taking into account that the a axis is tilted by 30 degrees in our optical setup. These observations confirm that the birefringence pattern arises from the presence of three orthorhombic domains in the sample [1].

FERMI SURFACE CALCULATIONS

Fermi surfaces (FS) are calculated by the Vienna Ab initio Simulation Package (VASP). Figure S5 shows the FS for $NdRu_3Si_2$ and $NdRu_3B_2$ in the pristine hexagonal and low-temperature ortho-III phase. In the case of $NdRu_3Si_2$, we see four flat sheets, which may indicate nesting along the k_z direction. Counting the number N_{FS} of flat FS sheets in the hexagonal and ortho-III phases, we find $N_{FS}(\text{ortho} - \text{III}) = 4N_{FS}(\text{hex.})$. Since the volume of the unit cell is 4 times larger in ortho-III than in the hexagonal structure, this excludes a nesting origin for the structural phase

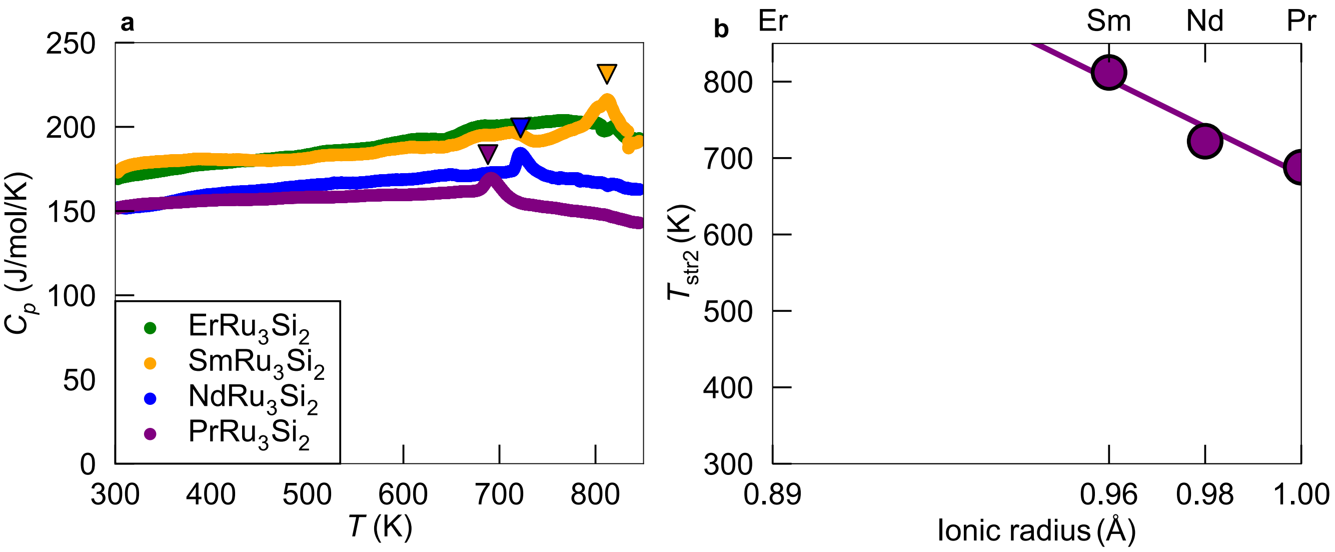

FIG. S3. Structural transition temperature ($T_{\rm str2}$) for $R\mathrm{Ru_3Si_2}$ (R=Pr, Nd, Sm, Er). **a**, Specific heat calculated from differential scanning calorimetry. **b**, Evolution of $T_{\rm str2}$ where the structural phase transition appears in the specific heat.

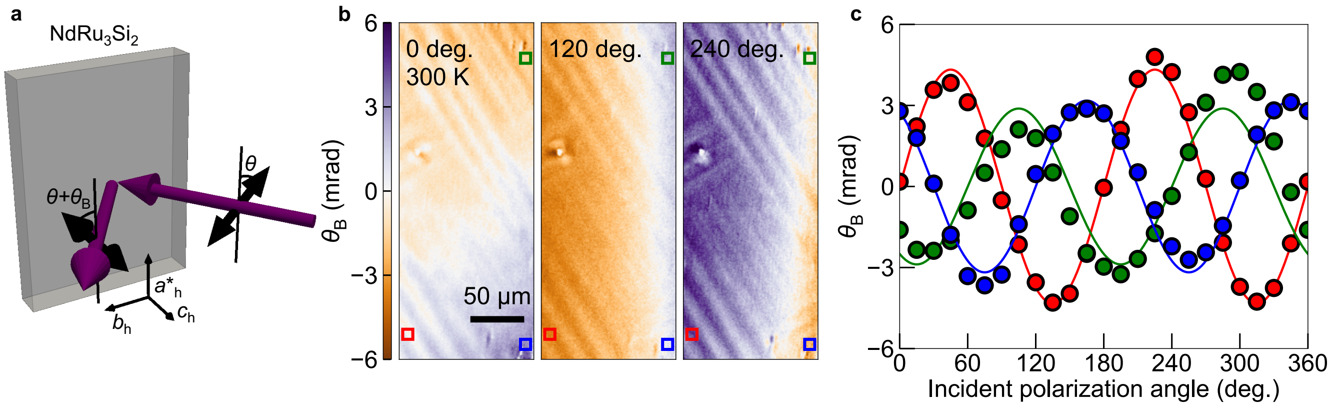

FIG. S4. Real-space observation of three orthorhombic domains by the optical birefringence measurement in $\mathrm{NdRu_3Si_2}$ at room temperature. **a**, Measurement geometry for the optical birefringence effect. θ and $\theta_{\rm B}$ denote the incident polarization angle and the polarization rotation angle, respectively. The a^* axis corresponds to $\theta = 0$. **b**, Spatial map of $\theta_{\rm B}$ measured with incident polarization angles of 0, 120, and 240 degrees. **c**, Incident polarization angle dependence of $\theta_{\rm B}$ for the three regions highlighted in panel **b**, consistent with the formation of three orthorhombic domains rotated by 120 degrees with respect to each other. Colors of filled circles correspond to those of regions in panel **b**.

transition. Instead of the nesting picture, which emphasizes the role of states in the close vicinity of the Fermi energy, the present molecular orbital scenario emphasizes the reconstruction of electronic states over a much larger energy window.

PARTIAL DENSITY OF STATES OF RU-$4d_z^2$ AND MAIN-GROUP p ORBITALS

Figure S6 shows the partial density of states (PDOS) of Ru-d_z^2 and Si-/B-p_z orbitals for $\mathrm{NdRu_3Si_2}$ and $\mathrm{NdRu_3B_2}$ in the hexagonal structure, respectively. As explained in the main text, the close electronegativity of Ru and B favors hybridization and pushes the PDOS of Ru-d_z^2 well above the Fermi level.

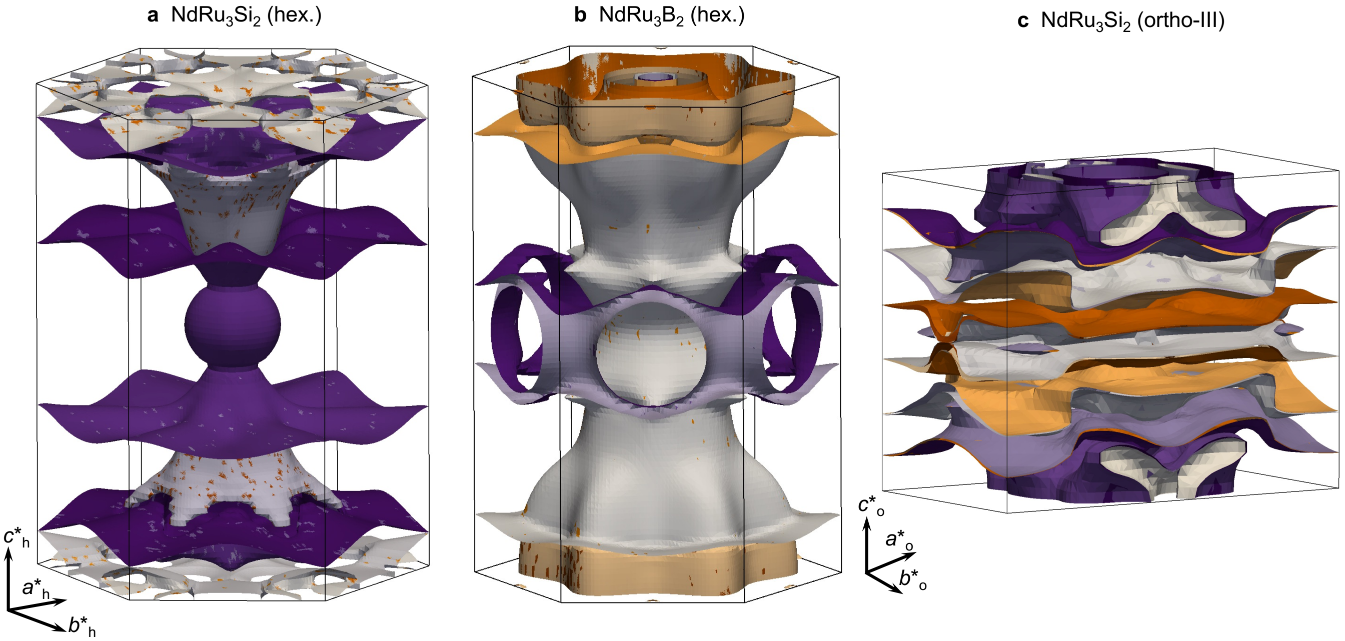

FIG. S5. **a**,**b**, Fermi surfaces of $NdRu_3Si_2$ and $NdRu_3B_2$ in the hexagonal structure, and **c** that of $NdRu_3Si_2$ in ortho-III.

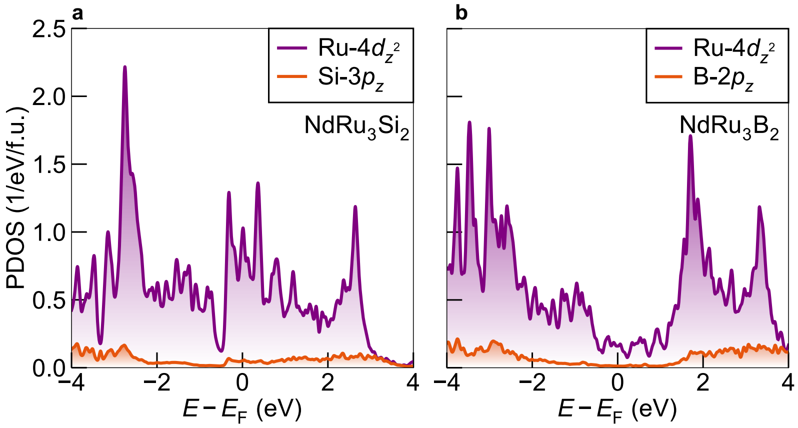

FIG. S6. **a**,**b**, Partial density of states (PDOS) of Ru-d_z^2 and Si-/B-p_z orbitals for $NdRu_3Si_2$ and $NdRu_3B_2$ in the hexagonal structure, respectively. For $NdRu_3B_2$, PDOS peaks of Ru-d_z^2, hybridized with unoccupied B-p_z orbitals, are located well above the Fermi level.

BAND STRUCTURE CALCULATIONS FOR $NDRU_3SI_2$ AND $NDRU_3B_2$

Figure S7 compares the band structures of the hexagonal and ortho-III phases. Since the orthorhombic structure has a unit cell four times larger than the hexagonal structure, the number of bands increases accordingly. In this case, the bands in the hexagonal $k_z = \pi$ plane are folded onto the orthorhombic $k_z = 0$ plane as a result of the doubling of the c axis. Therefore, the Γ–K and A–H directions in the hexagonal phase correspond to the Γ–X path. The fat bands near −0.3 eV split after the transition, in line with the formation of molecular orbitals.

In $NdRu_3B_2$, the van Hove singularity appears at the M point [Fig. 1**e**], as expected from the single-orbital tight-binding model. A similar feature is found at the A point, but this is likely coincidental rather than intrinsic to the kagome lattice, since the A point lies at $k_z = \pi$ and the dispersion there originates from interlayer hopping.

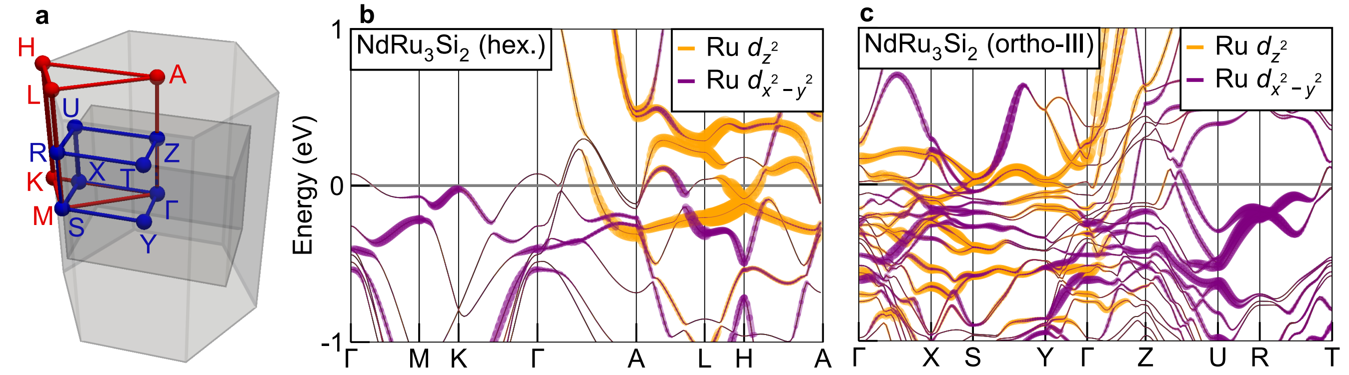

FIG. S7. **a**, Hexagonal (red) and primitive orthorhombic (blue) Brillouin zone. **b**,**c**, Band structures of $NdRu_3Si_2$ for the hexagonal and ortho-III structures, respectively.

APPLICABILITY OF THE PROPOSED FRAMEWORK: BEYOND 1-3-2.

Here, we summarize our argument on the orthorhombic structural instabilities of the kagome lattice observed in our study and discuss the applicability of the proposed framework to various kagome metals beyond the 1-3-2 family. **Applicability criteria.** Interlayer kagome dimerization is expected to occur when the following conditions are satisfied:

1. *Electronic condition:* states with appreciable transition-metal d_{z^2} weight lie near E_F, enabling interlayer metal–metal bonding across kagome planes. This tendency can be gauged by the electronegativity difference between the transition metal and the spacer atom, typically a main-group element, as proposed in this study.

2. *Geometric condition:* the shortest interlayer separation occurs between consecutive kagome layers. Some families, as shown below, exhibit the shortest interlayer distance between triangular layers, which promotes structural distortions between them.

Placement of major kagome families within this framework. Figure S8 provides a unified description of representative kagome families, which can be viewed as distinct stackings of triangular, honeycomb, and kagome lattices. We also indicate the characteristic structural instabilities associated with each family in Fig. S9.

1–3–2 (RT_3M_2): Kagome planes are relatively close and electronically active, fulfilling both electronic and geometric criteria. This regime is exemplified by our Ru-based silicides, where interlayer dimerization of the kagome sublattice naturally explains the observed instability and its Bragg/diffuse scattering fingerprints.

1–6–6 (MT_6Z_6): Two kagome layers per unit cell are separated by spacer networks, with the shortest distance instead connecting the triangular M–Z layers. Reported transitions are typically characterized by displacements along one-dimensional M–Z chains, bypassing the kagome T sites. Here, the geometric criterion disfavors kagome dimerization, and chain-driven instabilities dominate. Our mechanism is therefore not expected to be generic in this family unless specific chemistries shorten the effective kagome–kagome separation.

1–1 (e.g., FeGe): Fe sites form kagome layers while Ge supplies triangular/honeycomb layers. With comparable separations between kagome and triangular planes, both instabilities are possible. In FeGe, diffuse scattering near the ordering temperature has been modelled by interlayer dimerization on the triangular sublattice. Notably, the electronegativity difference between Fe ($\chi = 1.83$) and Ge ($\chi = 2.01$) is $\Delta\chi = 0.18$, similar to $\Delta\chi = 0.2$ for Ru–B. This small difference disfavors d_{z^2} hybridization between Fe atoms on the kagome lattice, consistent with our framework. Instead, the triangular-layer channel dominates, likely aided by the extended $4p$ orbitals of Ge. This family remains sparse, and further compounds would provide valuable tests of our criteria.

1–3–5 (e.g., AV_3Sb_5): Kagome planes are well separated by three spacer layers, shifting the balance toward in-plane distortions. Nonetheless, the observation of superlattice reflections in $CsCr_3Sb_5$ at the same wave vectors as in $LaRu_3Si_2$ indicates that, when electronic and geometric conditions partially align, aspects of the interlayer dimer phenomenology may still emerge.

Qualitative experimental expectations. For kagome metals that meet the above criteria, the following signatures are anticipated:

- *Bragg reflections:* superlattice reflections at L = half-integer planes due to doubling along c, with intensities dominated by the transition-metal form factor and systematic absences consistent with a dimer pattern linking adjacent kagome planes.
- *Diffuse scattering:* above the transition, anisotropic streaks along b^* with systematic absence in H = even and L = integer planes, reflecting the kagome geometry; upon cooling, these condense into superlattice Bragg peaks, as observed in $R\mathrm{Ru}_3\mathrm{Si}_2$.
- *Phonon/electronic signatures:* softening of $k_z = \pi$ modes involving out-of-plane displacements of kagome atoms, and partial lifting/splitting of kagome-derived d_{z^2} states near E_F, consistent with interlayer bonding–antibonding formation.

Implications for kagome metals beyond 1–3–2**.** The structural transitions in the 1–1 and 1–6–6 families are generally characterized by a one-dimensional chain between triangular layers, as illustrated in Fig. S9. Our dimerization mechanism thus provides a unifying framework that links these instabilities with kagome-driven ones, offering new insights into the broader problem of structural instabilities in kagome metals.

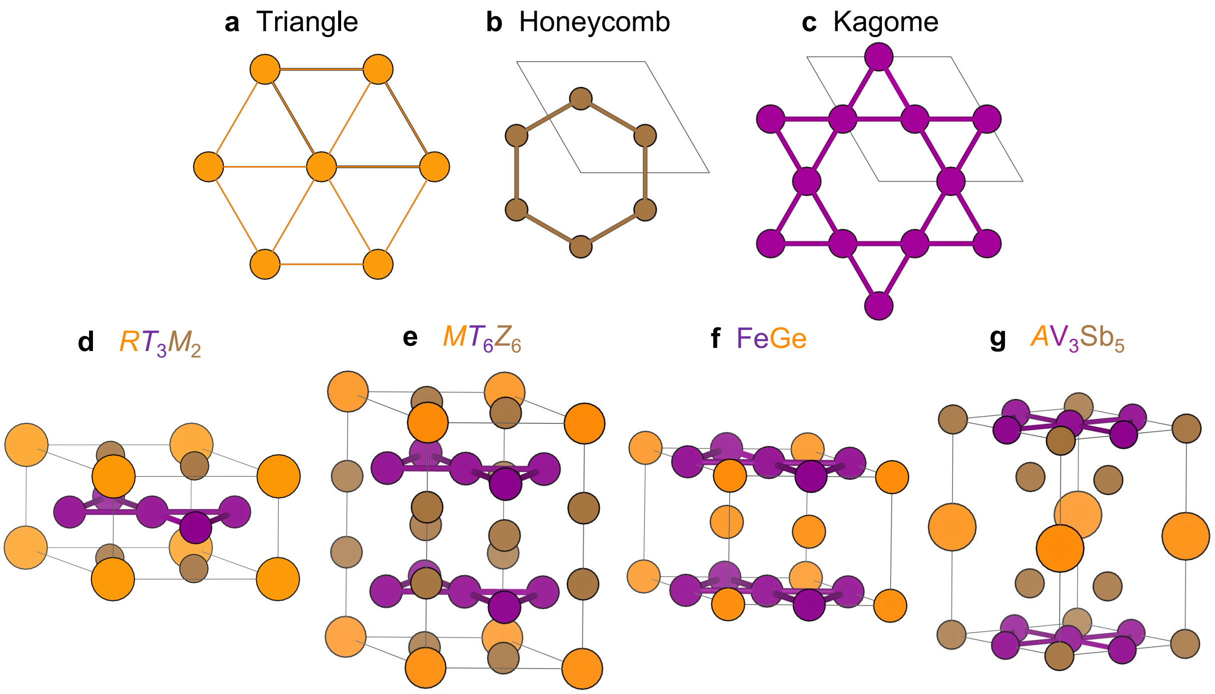

FIG. S8. Unified description of crystal structures of kagome metals. Distinct stacking patterns of the **a** triangle, **b** honeycomb, and **c** kagome lattice result in **d** 1-3-2, **e** 1-6-6, **f** 1-1, and **g** 1-3-5. In the case of 1-6-6, 1-1, and 1-3-5 families, a main-group element occupies both the honeycomb and triangular lattices.

DIFFUSE SCATTERING IN $R\mathrm{RU}_3\mathrm{SI}_2$

Using our charge model for kagome dimers, we illustrate three orthorhombic structures of $R\mathrm{Ru}_3\mathrm{Si}_2$ in Fig. S10. ortho-I and ortho-II are related by flipping charge signs on one of the triangles. In these structures, the central atom remains undimerized. On the other hand, ortho-III is characterized by dimerization of Ru_3 and undimerization of Ru_1 or Ru_2.

In Fig. S11, we simulate the diffraction pattern in the $L = 1.5$ plane for representative points p in the phase diagram of orthorhombic instabilities shown in Fig. 2**i**. When $p = 0.5, 0.2$, we successfully reproduce the diffuse scattering from

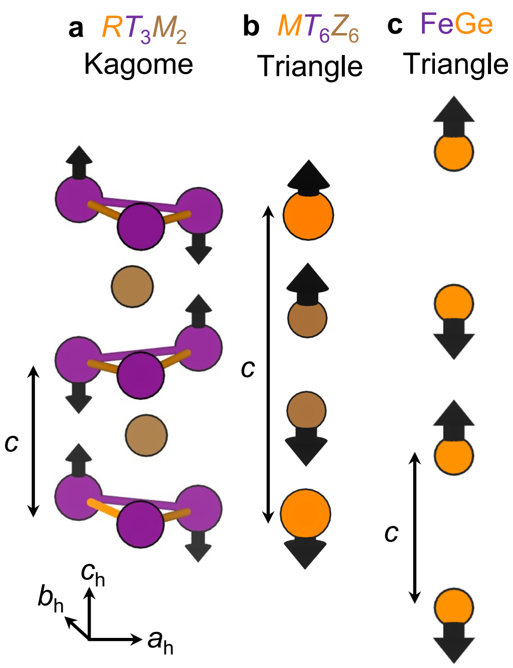

FIG. S9. Typical structural instabilities in kagome metals. **a**, Interlayer dimerization between kagome layers observed in RT_3M_2. **b**, Displacements along the M-Z chain between triangle lattices in MT_6Z_6. **c**, Interlayer dimerization between triangle layers in FeGe. While the distance between triangular layers and that between kagome layers is the same in the 1-1 family, FeGe preferentially dimerizes between triangular layers, unlike the RT_3M_2 compounds.

the hexagonal and ortho-I ($Cccm$) phases in $LaRu_3Si_2$, respectively [2]. Here, we set a $L_x \times L_y \times L_z = 30 \times 30 \times 1$ supercell, calculate the diffraction intensities on randomly sampled $10 \times 10 \times 1$ supercells, and average on 50 samples. The Ru displacement is set to $\delta = 0.0226$, the same value used in the simulation presented in the main text.

We also comment on the diffuse scattering between $T_{\rm str2}$ and 660 K. From the condition (ii) mentioned in the main text, two sublattices related by 1/2-translation along the $c_{\rm o}$ or $a_{\rm o}$ axis have to be displaced in opposite directions. However, this requirement is not satisfied in ortho-III [Fig. S10**e**,**f**]: Ru_1 and Ru_2 are no longer symmetry-equivalent and one of them is undimerized, as opposed to ortho-I and ortho-II. Hence, the diffuse scattering in this temperature range is attributed to remnants of the short-range correlated structure above $T_{\rm str2}$, which gradually decreases as ortho-III stabilizes. We also note that Ru_3 does not contribute to the diffuse scattering. Ru_3 occupies the corners of the unit cell, as shown in Fig. S10**f**, so once the average structure is fixed, its displacement is uniquely determined. The same argument applies to the Nd atoms, which reside at the corners [Fig. 1**a**].

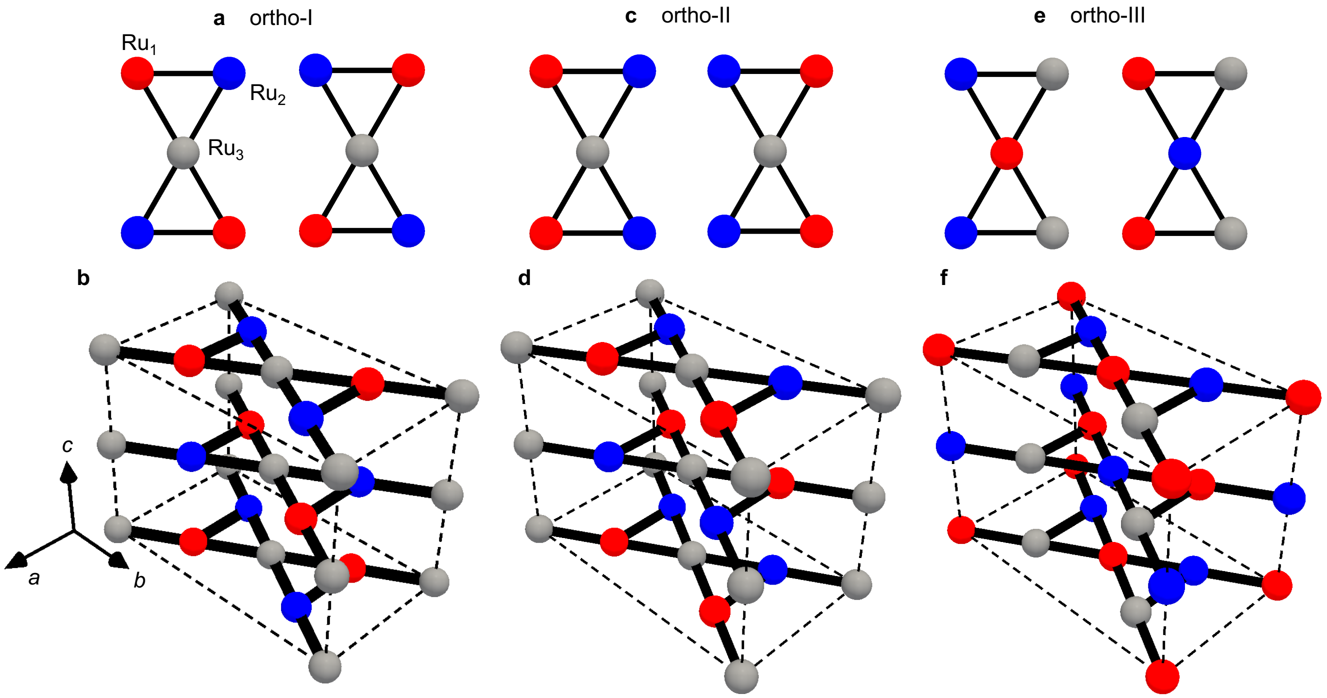

FIG. S10. Distorted kagome structures obtained from the charge model. **a**,**b** ortho-I ($Cccm$) realized in $LaRu_3Si_2$. **c**,**d** ortho-II ($Ibmm$) realized as short-ranged ordered phase in $NdRu_3Si_2$. The structure is related to ortho-I by flipping charge signs on one of the two triangles. **e**,**f** ortho-III ($Pbmm$) realized in $NdRu_3Si_2$ at low-temperatures. In contrast to the other two, Ru_3 is charged (dimerized).

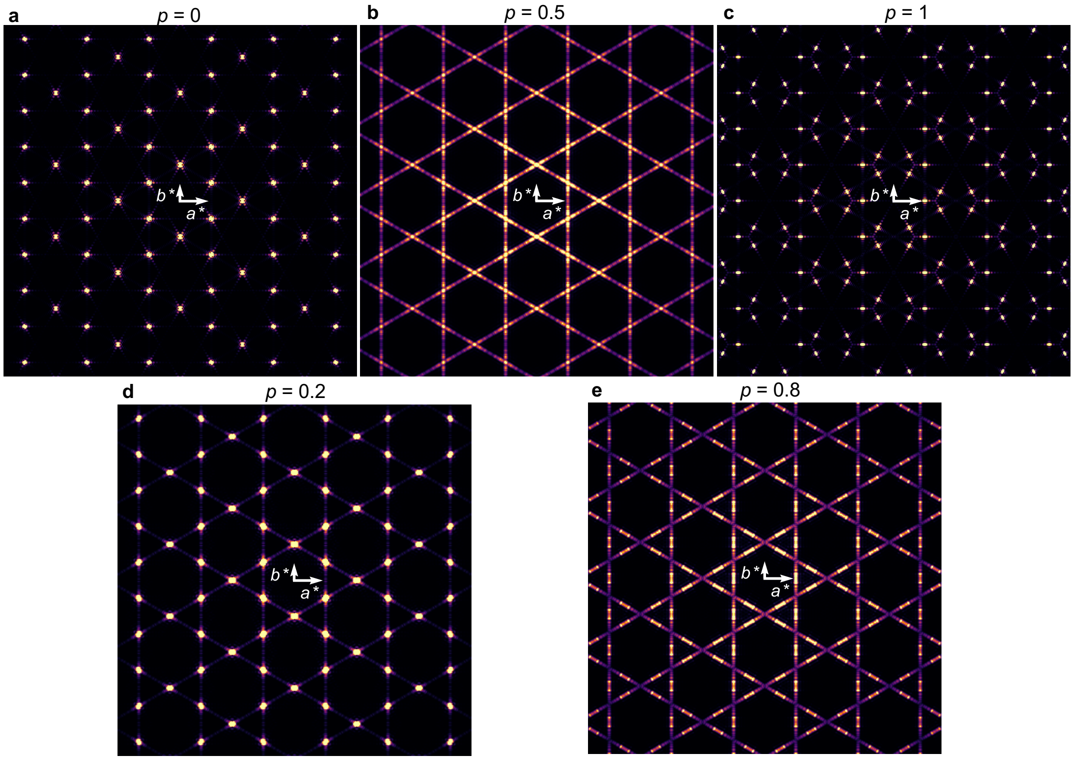

FIG. S11. Simulated diffuse scattering in the $L = 1.5$ plane for representative probability values p: **a**, $p = 0$ (ortho-I), **b**, $p = 0.5$ (disorder), **c**, $p = 1$ (ortho-II), **d**, $p = 0.2$ (short-ranged ortho-I), **e**, $p = 0.8$ (short-ranged ortho-II).

* misawann6@g.ecc.u-tokyo.ac.jp
† hirschberger@ap.t.u-tokyo.ac.jp

[1] Y. Xu, Z. Ni, Y. Liu, B. R. Ortiz, Q. Deng, S. D. Wilson, B. Yan, L. Balents, and L. Wu, Nat. Phys. **18**, 1470 (2022).
[2] I. Plokhikh, C. Mielke, III, H. Nakamura, V. Petricek, Y. Qin, V. Sazgari, J. Küspert, I. Biało, S. Shin, O. Ivashko, J. N. Graham, M. v. Zimmermann, M. Medarde, A. Amato, R. Khasanov, H. Luetkens, M. H. Fischer, M. Z. Hasan, J.-X. Yin, T. Neupert, J. Chang, G. Xu, S. Nakatsuji, E. Pomjakushina, D. J. Gawryluk, and Z. Guguchia, Commun. Phys. **7**, 1 (2024).
